# The Effects of Salinity on the Anatomy and Gene Expression Patterns in Leaflets of Tomato cv. Micro-Tom

**DOI:** 10.3390/genes12081165

**Published:** 2021-07-29

**Authors:** Jonas Hoffmann, Roberto Berni, Flavia Maria Sutera, Annelie Gutsch, Jean-Francois Hausman, Suzanne Saffie-Siebert, Gea Guerriero

**Affiliations:** 1 Environmental Research and Innovation Department, Luxembourg Institute of Science and Technology, 5, rue Bommel, L-4940 Hautcharage, Luxembourg; jonashoffmann@hotmail.be (J.H.); annelie.gutsch@list.lu (A.G.); jean-francois.hausman@list.lu (J.-F.H.); 2 TERRA Teaching and Research Center, Gembloux Agro-Bio Tech, University of Liège, 5030 Gembloux, Belgium; Roberto.Berni@uliege.be; 3 SiSaf Ltd., Surrey Research Park, Guildford GU2 7RE, UK; sutera@sisaf.com (F.M.S.); saffie@sisaf.com (S.S.-S.)

**Keywords:** tomato cultivar Micro-Tom, salinity, qPCR, microscopy, leaflets, cell wall, phenylpropanoid pathway

## Abstract

Salinity is a form of abiotic stress that impacts growth and development in several economically relevant crops and is a top-ranking threat to agriculture, considering the average rise in the sea level caused by global warming. Tomato is moderately sensitive to salinity and shows adaptive mechanisms to this abiotic stressor. A case study on the dwarf tomato model Micro-Tom is here presented in which the response to salt stress (NaCl 200 mM) was investigated to shed light on the changes occurring at the expression level in genes involved in cell wall-related processes, phenylpropanoid pathway, stress response, volatiles’ emission and secondary metabolites’ production. In particular, the response was analyzed by sampling older/younger leaflets positioned at different stem heights (top and bottom of the stem) and locations along the rachis (terminal and lateral) with the goal of identifying the most responsive one(s). Tomato plants cv. Micro-Tom responded to increasing concentrations of NaCl (0-100-200-400 mM) by reducing the leaf biomass, stem diameter and height. Microscopy revealed stronger effects on leaves sampled at the bottom and the expression analysis identified clusters of genes expressed preferentially in older or younger leaflets. Stress-related genes displayed a stronger induction in lateral leaflets sampled at the bottom. In conclusion, in tomato cv. Micro-Tom subjected to salt stress, the bottom leaflets showed stronger stress signs and response, while top leaflets were less impacted by the abiotic stressor and had an increased expression of cell wall-related genes involved in expansion.

## 1. Introduction

Salinity is an abiotic stressor that severely affects plant growth, development and reduces the final biomass yield, as well as fruits’ number and weight [1]. With respect to their tolerance to salt stress, plants are divided into halophytes and glycophytes (terms referring to plants that can grow or not on saline soils) which differ both in biochemical mechanisms (ion compartmentalization, osmolytes’ production, enzymatic activities [2]) and in specialized anatomical structures (namely salt glands [3]).

Cultivated tomato (*Solanum lycopersicum* L.) is a glycophyte that is moderately sensitive to salinity [4,5]. The study of the response of tomato to salinity has important implications, since soil salinization is becoming a major threat to agriculture, especially in coastal areas that are normally more prone to it [6]. In the literature, several papers have demonstrated the molecular mechanisms of cultivated tomato and its wild relatives (e.g., *Solanum chilense*) in response to salinity with the goal of identifying key players involved in stress tolerance. For example, genes coding for ethylene-response factors (ERFs) were highly induced in *S. chilense* upon salt stress [7], as well as a transcript involved in vesicular trafficking [8] and in the Wnt (Wingless-related integration site) signaling pathway [9] which relies on cell surface receptors. These data are relevant to devise breeding strategies aimed at increasing the tolerance to salinity in cultivated tomato.

Gathering molecular information on the response of tomato leaves to salinity is relevant to understand how the changes in gene expression correlate with modifications in physiological parameters, such as photosynthesis, stomatal conductance, osmolytes’ and biomass accumulation, primary/secondary metabolites’ biosynthesis. Previously, a study investigated the expression of genes involved in cytokinin biosynthesis, i.e., two isopentenyltransferases (*SlIPT3* and *SlIPT4*), in leaves, stems and roots of tomato plants exposed to salt stress and revealed that the transcripts accumulated to a different extent in older and younger leaves of control plants and responded to the abiotic stressor [10]. In another investigation, the response to cytokinin application of tomato old and young leaves was studied using RNA-Seq and revealed that, in younger leaves, genes involved in transcription, translation, cell division and signal transduction were highly expressed, while, in older ones, transcripts involved in stress and defense response, protein degradation, as well as encoding receptor-like kinases and cytochrome P450 were more abundant [11].

Concerning the concentrations of salt used on tomato, studies have focused on the range 50–450 mM. For example, Bacha and colleagues used concentrations of 50 and 150 mM NaCl on tomato cv. Micro-Tom [12]; studies on the same cultivar and aiming to evaluate stress alleviation with plant growth promoting bacteria used a concentration of 180 mM [13,14] and, finally, Al Hassan and colleagues used higher concentrations on cherry tomatoes (150, 300 and 450 mM) to evaluate salt stress response [15].

The leaves of tomato are compound and composed of leaflets, each one resembling a simple leaf attached to the central rachis. A previous study showed that cytokinin plays a role in determining the compound leaf architecture in tomato by maintaining the activity of the leaf marginal blastozone through a regulation that takes place downstream of KNOX1 proteins and that this effect depends on the proper localization of auxin maxima (i.e., the balance of both auxin and cytokinin is responsible for the leaf shape) [16].

A useful model for molecular studies in tomato is the dwarf cultivar (cv.) Micro-Tom [17], which has a small size and a short life-cycle of 70–90 days. Previous investigations addressed its response mechanisms to salinity: it was observed that tomato cv. Micro-Tom decreased the leaf area and modulated transpiration after salt application, that the photosynthetic rate was reduced and the content of total phenolics increased [12]. RNA-Seq identified more than 4600 differentially expressed genes following the addition of 150 mM NaCl, among which cytokinin-related transcripts which correlated with the accumulation of several active forms of the phytohormone after salt stress [18].

The present study investigated the response of tomato cv. Micro-Tom (hereafter referred to as Micro-Tom) lateral and terminal leaflets sampled at the top and bottom of the stem. The goal was to identify the most responsive leaflet to salt stress for future molecular studies focused on this tomato model. An approach combining light microscopy with targeted gene expression analysis (qPCR) was adopted.

## 2. Materials and Methods

### 2.1. Experimental Set-Up and Growth Conditions

Micro-tom seeds (https://www.graines-baumaux.fr/170325-tomate-micro-tom.html) were sown in pots (size: 10 cm × 10 cm × 12 cm) filled with soil composed of a mixture of compost from peat, perlite, NPK and magnesium fertilizers (COMPO, Aalter, Belgium) and sand (Agricon, Balen, Belgium) (1:1 *w*/*w*). After 10 days, the seedlings were separated and put in single pots containing the same soil mixture. Each pot stood on a plate to avoid water sharing between plants. Plants were then grown during the whole experiment in a climatic chamber (Fitotron, Weiss Technik, Reiskirchen, Germany) (23 °C, 60% relative humidity-RH, 16/8 h photoperiod). Salt treatment was applied from the 10th day after sprouting till the end of the experiment. The application of the salt stressor was performed by solubilizing a proper amount of NaCl in tap water, in order to simulate alteration of the watering source. Plants were watered 2 to 3 times a week with the same amount of tap water with and without NaCl. In order to determine the effects of salinity on Micro-Tom leaflets and stem tissues, an increasing range of salt concentration (0, 100, 200 and 400 mM) was applied to plants. These concentrations were determined according to the literature [12,13,14].

To perform the microscopy analysis and identify the effects of increasing salt concentrations on the bottom/top leaflets and stem tissues, NaCl at 100, 200 and 400 mM was tested. Two plants were exposed to each concentration, while three plants were used as control. Height and stem diameter measurements (performed with a digital caliper) were done two to three times a week and the positions of the plants were randomized in the incubators to avoid any bias. Leaves and stems were sampled for microscopy analysis in 4-week-old plants.

To determine the genes’ response to salinity in leaflets sampled at different height and positions along the rachis, a concentration of 200 mM NaCl was used. Twelve plants were watered with NaCl 200 mM dissolved in tap water, while another twelve were watered with tap water for 17 days. These plants were divided into three biological replicates, so that each replicate was composed of a pool of four plants. Sampling was performed when plants were 4 weeks old and according to Figure S1.

Stem diameter and height were measured together with the fresh weight (FW) of aerial parts (stems plus leaflets) and roots. Dry weight (DW) was measured after drying the tissues at room temperature for 2 weeks.

### 2.2. Sample Preparation and Microscopy

Leaves and stems of 4-week-old plants were sampled and cut transversally before being embedded in 5% (*w*/*v*) low melting point agarose solution (Sigma-Aldrich, Merck, Darmstadt, Germany). Cross sections of 100 µm were cut using a vibratome (Leica Biosystems, Nussloch, Germany) and were directly observed under the microscope (Olympus BX51, Tokyo, Japan) which was equipped with a Toupview camera and software (ToupTek Photonics, Zhejiang, China).

Five mm thick/long segments/sections of the leaves were dipped for 24 h at 4 °C in fixative solution composed of 50 mL of phosphate buffer 400 mM pH 7.2 (final concentration 200 mM), 20 mL paraformaldehyde 10% (*w*/*v*) (final percentage 2% *w*/*v*), 2 mL glutaraldehyde 50% (*v*/*v*) (final percentage 1% *v*/*v*), 1 g of caffeine (final percentage 1% *w*/*v*) and 28 mL dH_2_O. For optimal fixation, samples were subjected to vacuum for 15 min. The leaflet samples were then transferred in 70% (*v*/*v*) ethanol and stored at 4 °C until further processing. The next steps consisted in the dehydration of tissues with successive baths in increasing ethanol concentrations (*v*/*v*, 95% for 30 min, 95% for 1 h, 100% for 30 min). After complete dehydration, tissues were transferred for 2 h into a 1:1 (*v*/*v*) solution of ethanol 100% and a soaking solution composed of 100 mL resin (Technovit 7100, Kulzer Technik, Wehrheim, Germany), 1 g of hardener in powder from the kit and 2 mL of polyethylene glycol 400 (Sigma-Aldrich). Subsequently, samples were transferred for 24 h at 4 °C in 100% soaking solution before moulding into histomold (Leica Biosystems, Nussloch, Germany) with hardening solution (Technovit 7100, Kulzer Technik) added to the soaking solution (1:15 (*v*/*v*)). Moulds were then placed in an oven at 37 °C until complete hardening of the resin (3 days). Ten µm cross sections were cut using a microtome (Leica Biosystems, Nussloch, Germany) and observed under the microscope.

The determination of the xylem vessels’ thickness and area, as well as of the intercellular spaces was performed with the open-source ImageJ/Fiji software: ImageJ 2.0.0/1.53c/Java 1.8.0_172 (64-bit) [19] by measuring 20–25 cell types.

### 2.3. Design and Validation of Primers

Primers were designed using Primer3Plus with qPCR parameters [20] and verified with the OligoAnalyzer tool from Integrated DNA Technologies (https://eu.idtdna.com/calc/analyzer) (Coralville, IA, USA). The coding sequences were obtained using Phytozome12 (https://phytozome.jgi.doe.gov/pz/portal.html#) or Genbank (https://www.ncbi.nlm.nih.gov/gene/). Only genes showing expression in leaves of tomato (analysis carried out using the eFP browser; available at http://bar.utoronto.ca/efp_tomato/cgi-bin/efpWeb.cgi) were retained for primer design. The specificity of the primers on redundant families of genes was evaluated by sequence alignment with ClustalOmega (EMBL-EBI) after a BLAST (Basic Local Alignment Search Tool) search on Phytozome12 of the target sequence. Genes with the lowest e-value were selected for BLAST and aligned.

Primer efficiencies were determined by qPCR using serial five-fold dilutions (10, 2, 0.4, 0.08, 0.016, 0.0032 ng/µL) of cDNA obtained from a pool of tissues from each salt stress condition. R^2^ and amplification efficiencies (whereby 100% is defined as 2) were calculated using QuantStudio™ Design & Analysis Software v1.5.1 (Fisher Scientific, Merelbeke, Belgium) after discarding the outliers. The list and details of the primers are shown in Table S1.

### 2.4. RNA Extraction, cDNA Synthesis and qPCR

Leaflets were sampled and immediately flash frozen in liquid nitrogen for storage at −80 °C until further processing. They were then ground to a fine powder with autoclaved steel beads in a Mixer-mill MM 400 (Retsch, Haan, Germany) for 2 min at 24 Hz with frozen plastic supports for tubes (i.e., previously plunged in liquid nitrogen) to avoid sample thawing. Samples were processed for RNA extraction without delay. RNA extraction was carried out with the RNeasy Mini Kit (Qiagen, Leusden, The Netherlands) after homogenization of tissue lysates with the QIAshredder Kit (Qiagen). Kits were used following the manufacturer’s instructions for total plant RNA extraction with a supplementary on-column DNase I purification step (Qiagen) to avoid DNA contamination. RNA was finally eluted using RNase-free water. RNA concentrations were determined with NanoPhotometer NP80 (Implen, Munich, Germany) with RNase-free water as blank. Samples with concentrations lower than 100 ng/µL were re-extracted, while samples with A260/230 < 2 were cleaned-up by RNA precipitation with ammonium acetate (NH_4_OAc) as described in [21]. The RNA Integrity Number (RIN) was evaluated by capillary gel electrophoresis with a 2100 Bioanalyzer (Agilent, Santa Clara, CA, USA) according to the manufacturer’s instructions, using the RNA 6000 nano chip (Agilent). RNA (1 µg) was retro-transcribed and the cDNA diluted to 2 ng/µL. qPCR analyses were carried out with Takyon Low ROX SYBR Green (Eurogentec, Liege, Belgium) in 384 wells reaction plates using 10 µL reaction volume. Plates were filled using an automated dispensing device (epMotion 5073x, Eppendorf, Hambourg, Germany) for optimal reproducibility, with 3 technical replicates for each sample. A melt-curve analysis was performed to check the presence of a single peak denoting specific amplification.

### 2.5. Gene Expression Analysis and Statistics

Gene expression was determined using qBase^PLUS^ (Biogazelle, Ghent, Belgium) [22] with the implemented geNORM tool that determined for both experiments, two reference genes as sufficient for data normalization (*Cyclophilin* and *AP2C*) among 4 tested (*FUL1*, *SAND*, *Cyclophilin* and *AP2C* [23]). The normalized relative quantities were log2-transformed and then the obtained values were used to perform statistics. Statistical analysis was carried out using IBM SPSS Statistics v20 (IBM SPSS, Chicago, IL, USA). Gene expression patterns were hierarchically clustered using Cluster 3.0 [24] and plotted as a heatmap with Java Treeview [25] (available at http://jtreeview.sourceforge.net/. Normality and homogeneity of the data were assessed using a Shapiro-Wilk and Levene’s test, respectively. A one-way ANOVA with a Tukey’s post-hoc test was used when parametric test was possible, while a Kruskal-Wallis with Dunn’s post-hoc test was used when parametric test’s conditions were not satisfied.

## 3. Results and Discussion

### 3.1. Effects of Increasing Salt Concentrations on Micro-Tom Leaves and Stems

**Figure 1:**
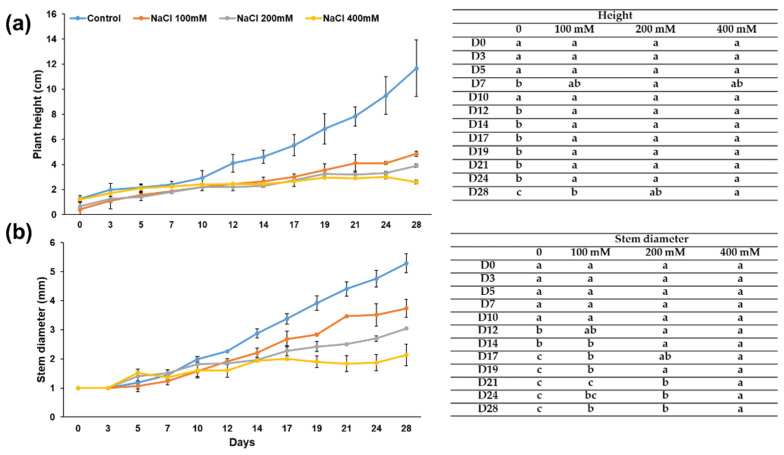
Impact of increasing concentrations of NaCl on Micro-Tom from day 0 to day 28. (**a**) Plant height and (**b**) stem thickness. Tables on the right-hand side: statistical significance of the data shown in Figure 1. Different letters denote statistically different values among groups at the ANOVA one-way with Tukey’s post-hoc test. “D”refers to days. A compact letter display [26] is used to indicate the significance between groups of samples. For height: D0 [F(3,5) = 2.45, *p* = 0.179], D3 [F(3,5) = 1.62, *p* = 0.296], D5 [F(3,5) = 4.87, *p* = 0.061], D7 [F(3,5) = 7.35, *p* = 0.028], D10 [F(3,5) = 2.55, *p* = 0.169], D12 [F(3,5) = 13.94, *p* = 0.007], D14 [F(3,5) = 27.71, *p* = 0.002], D17 [F(3,5) = 20.49, *p* = 0.003], D19 [F(3,5) = 21.18, *p* = 0.003], D21 [F(3,5) = 46.41, *p* = 0.000], D24 [F(3,5) = 63.56, *p* = 0.000], D28 [F(3,5) = 62.03, *p* = 0.000]. For stem thickness: D5 [F(3,5) = 3.78, *p* = 0.093], D7 [F(3,5) = 2.87, *p* = 0.143], D10 [F(3,5) = 1.79, *p* = 0.266], D12 [F(3,5) = 11.97, *p* = 0.010], D14 [F(3,5) = 46.48, *p* = 0.000], D17 [F(3,5) = 40.68, *p* = 0.001], D19 [F(3,5) = 58.57, *p* = 0.000], D21 [F(3,5) = 61.36, *p* = 0.000], D24 [F(3,5) = 43.47, *p* = 0.001], D28 [F(3,5) = 38.57, *p* = 0.001].

Micro-Tom plants grew slower upon salt stress and were smaller than the control plants watered with tap water (Figure 1). Plants subjected to 400 mM salt stress showed, as expected, the most severe symptoms: they stopped growing much earlier than the other plants treated with lower concentrations and were starting to wilt when sampled after four weeks of treatment.

**Figure 2:**
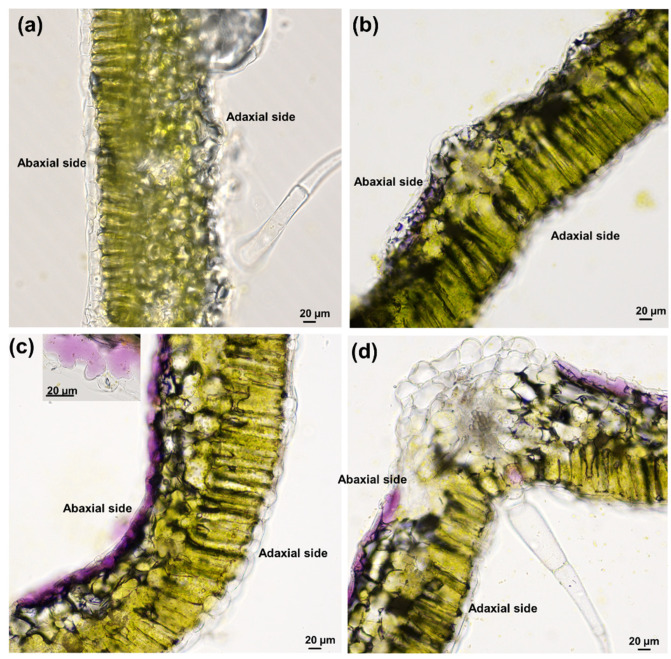
Vibratome section of a representative leaflet from (**a**) control; (**b**) 100 mM NaCl; (**c**) 200 mM NaCl; (**d**) 400 mM NaCl. A dark purple coloration appeared on the abaxial side of the NaCl-treated plants. Inset in c: detail of a jigsaw puzzle-like epidermal cells showing a purple color.

With an increasing salt concentration, a purple coloration started to develop rapidly in the epidermal layer of the abaxial side of leaves (Figure 2). Anthocyanins act in stress response by increasing the solute content in the vacuoles thereby lowering the osmotic potential of the leaf [27]. Additionally, it was shown that anthocyanins’ accumulation led to a metabolic shift in leaves with respect to amino acids and non-structural carbohydrates: proline content increased together with starch reserves’ use to provide carbon skeletons required for the synthesis of metabolites involved in osmotic adjustment [28].

**Figure 3:**
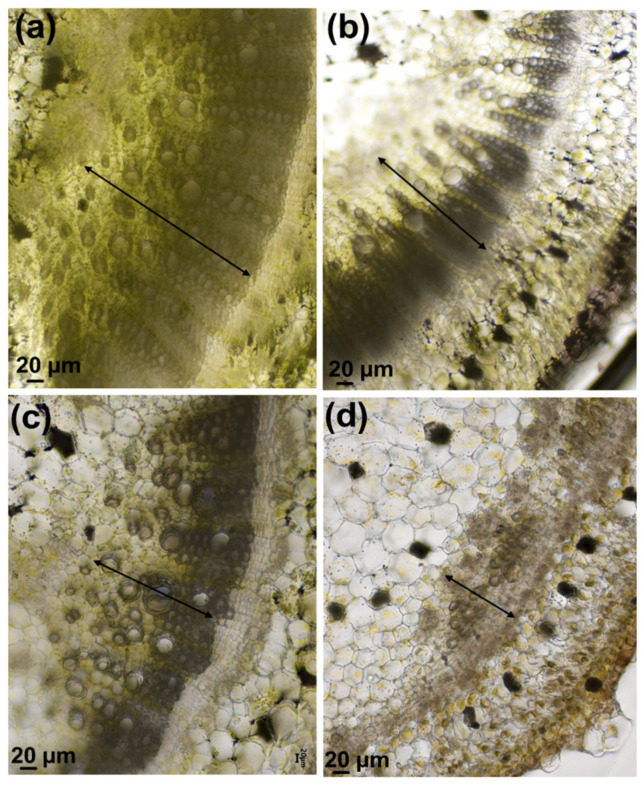
Vibratome cross sections of Micro-Tom stems subjected to increasing concentrations of NaCl. (**a**) control; (**b**) 100 mM NaCl; (**c**) 200 mM NaCl; (**d**) 400 mM NaCl. The double-headed arrow shows the xylem tissue.

Vibratome sections of Micro-Tom stems showed the effects of salt on the vascular tissue. Figure 3 shows indeed a less developed xylem tissue, an altered thickening of the vascular tissues and a reduction of the vessels’ diameter at the highest concentration.

The reduction of the vessels’ diameter is a common phenomenon among plants that decreases the incidence of cavitation [29]; the increased thickening of the xylem vessels improves stability by enhancing the mechanical properties of the secondary cell walls.

The stems showed a reduction of the xylem tissue, particularly evident for plants treated with 200 mM NaCl: this observation led to perform a microscopic assessment of the vascular bundle in the central veins of Micro-Tom leaflets at two different stem heights (bottom and top, corresponding to older and younger leaflets).

**Figure 4:**
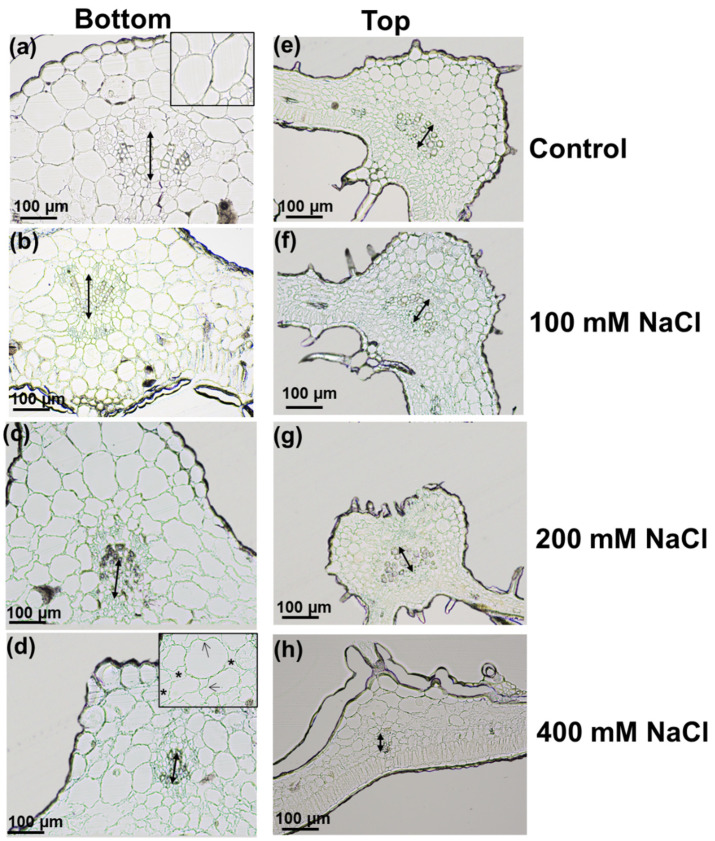
Microtome sections of bottom (**a**–**d**) and top (**e**–**h**) leaflets of Micro-Tom plants under control conditions and exposed to 100 mM, 200 mM, 400 mM NaCl. The region corresponding to the central vein is shown. The double-headed arrow shows the xylem. The asterisks and arrows in the inset in (**d**) refer to the intercellular spaces and cell walls’ invaginations.

The morphology of the leaflets was different between the bottom and the top of the plant, since those located at the top were smaller than the ones sampled at the bottom (Figure 4). As observed in the stems (Figure 3), the xylem vessels of bottom leaflets were less developed in plants exposed to increasing concentrations of NaCl: the xylem vessels had smaller lumens’ area at 400 mM compared to control or 100 mM NaCl-treated plants (Figure 4a–d and Figure S2a,c). The same effect was observed on hemp leaves, where the impact of salt stress was compared in young and older leaves. In *Cannabis sativa*, a reduced number and a smaller diameter of xylem vessels were indeed shown [30].

Measures of the xylem vessels’ cell wall thickness revealed non-significant changes (Figure S2b,d) between control and salt-stressed leaflets, although a significant increase was noticed when comparing 100 vs. 400 mM NaCl in bottom leaflets (Figure S2b). Sánchez-Aguayo and colleagues showed that salt stress enhanced xylem development by increasing lignification in vascular bundle with a gradually less evident effect in roots, stem and leaves, where leaves were the less affected organs [31].

Both collenchyma and parenchyma lost their regular contours with increasing salt concentrations since the cell walls showed invaginations, giving an overall wrinkly appearance that was particularly evident at 400 mM (see insets in Figure 4a and the arrows in the inset in d). Additionally, the intercellular spaces increased as a consequence of the cell walls’ invagination (asterisks in the inset in d).

In younger leaflets, stress effects were less severe than in older ones at the concentrations of 100 and 200 mM (Figure 4e–g), while narrower xylem vessels and a less developed xylem tissues were well visible in young leaflets exposed to 400 mM (Figure 4h). Younger leaflets have an active primary metabolism involved in expansion and growth, as well as an active reactive oxygen species-ROS scavenging activity counteracting the damages on membranes/lipids/nucleic acids. Older leaves that have ceased to expand and develop and start to senesce are the ones that will be impacted earlier by stress. A remobilization of carbon resources from older to younger leaves is also invoked as a strategy to increase resilience: this mechanism may therefore contribute to maintain the development of younger leaves under stress conditions [32].

**Figure 5:**
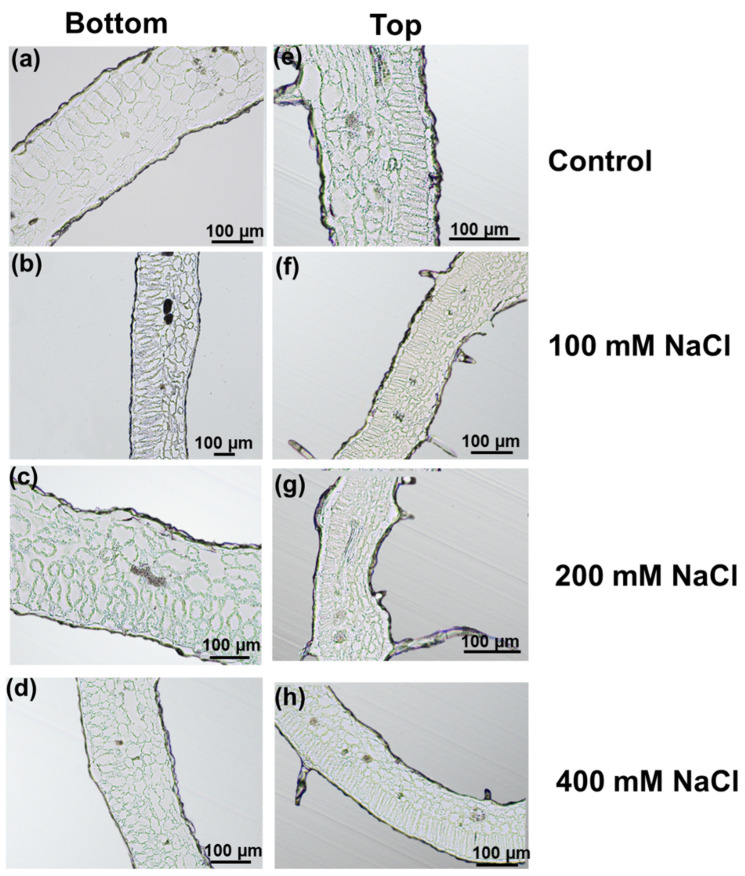
Microtome sections of bottom (**a**–**d**) and top (**e**–**h**) leaflets of Micro-Tom plants under control conditions and exposed to 100 mM, 200 mM, 400 mM NaCl. The palisade and spongy layers are shown.

The palisade layer in bottom leaflets was normally constituted by a single layer of elongated cells (Figure 5a). In the bottom leaflets under high salt levels (200 and 400 mM), the palisade cells were smaller and appeared stacked because of constrictions (Figure 5c,d). Under control conditions, the spongy parenchyma was characterized by scattered cells separated by air spaces (Figure 5a,e), but it became denser under stress conditions (Figure 5b–d and Figure S3), since the intercellular spaces decreased. A marked reduction in intercellular air spaces was also observed in *S. tuberosum* and *Arbutus unedo* exposed to NaCl [33,34]: the denser parenchyma affects gas exchanges within the mesophyll and ultimately impacts the conductance of CO_2_ with consequences on photosynthetic performance.

The palisade parenchyma of younger leaves (Figure 5e–h) was regular and tightly packed in a single row independently from the salt concentration, while the intercellular spaces in spongy mesophyll decreased with increasing salt concentration, similarly to bottom leaflets (Figure 5a–d).

**Figure 6:**
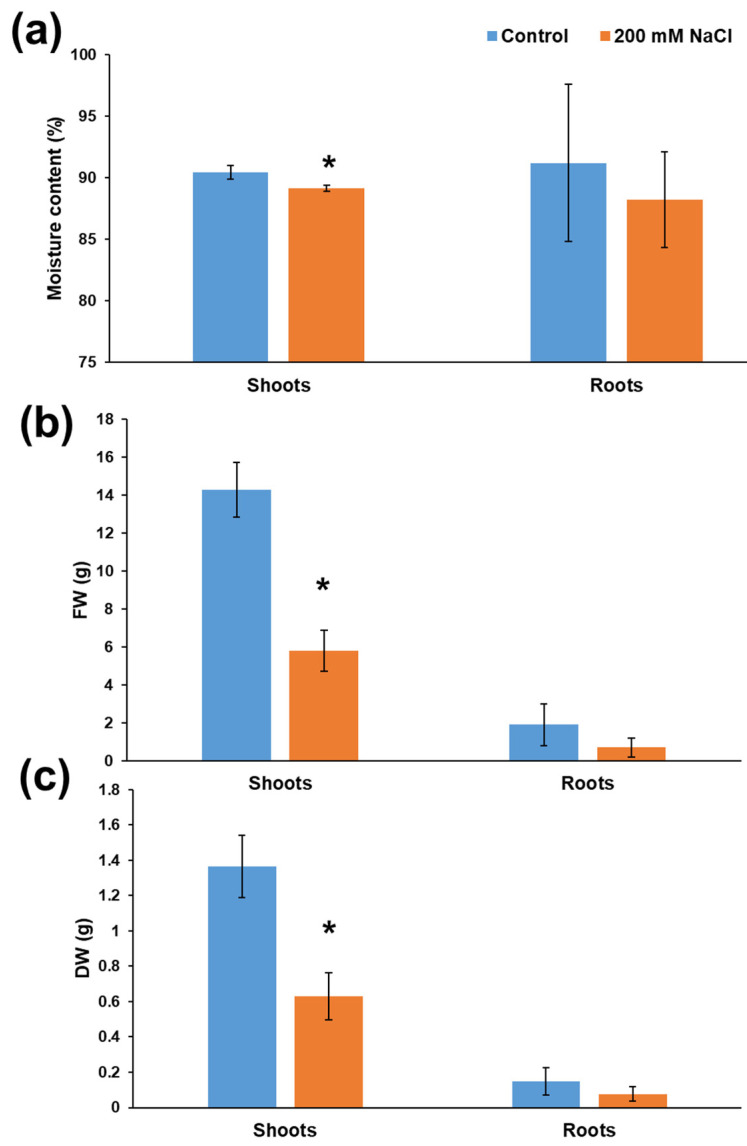
Basic biological parameters in control and salt-exposed Micro-Tom. (**a**) Moisture content (%); (**b**) fresh weight (FW) in g; (**c**) dry weight (DW) in g. Asterisks indicate statistically significant differences at the two-tailed *t*-test (* *p* < 0.05).

The results demonstrated that 200 mM NaCl was a concentration allowing the observation of differences in the growth and leaflets’ histology in the timeframe of four weeks. Other important biological parameters, namely the moisture content of shoots, the biomass of shoots and roots, decreased in stressed plants exposed to 200 mM NaCl (Figure 6). In the light of the differences observed in plants treated with 200 mM NaCl compared to control ones, this salt concentration was chosen for gene expression analysis. The condition of 200 mM NaCl was previously used for textile hemp [35] and the conductivity of the soil treated with this salt concentration was reported to be 5 mS cm^−1^ (0.8 for the control condition). Therefore, the salinity condition used corresponds to a slightly saline condition (4 < ECe < 8, as reported in [36]). As such, the concentration used allowed the study of the molecular response of Micro-Tom leaflets to a realistic condition of slightly saline soil environment.

### 3.2. Gene Expression Analysis in Leaflets of Micro-Tom Exposed to Salinity

For qPCR analysis, 46 genes involved in cell wall remodelling, emission of volatiles, phenylpropanoids’ biosynthesis and stress response were chosen, given their documented role in the response to environmental constraints [30,35,37,38]. The goal was to provide information on the expression dynamics for members involved in the biosynthesis/remodelling of cell walls (which are the first line of defense of plants to exogenous stresses), the synthesis of secondary metabolites and volatiles, whose abundance changes in response to stress [39,40]. Genes involved in stress response (e.g., coding for heat shock proteins, late embryogenesis abundant proteins) and ROS scavenging were also included to get an understanding of the transcriptional changes in the leaflets in response to salinity. The expression values were obtained for lateral and terminal leaflets sampled at the bottom and top of the stem.

**Figure 7:**
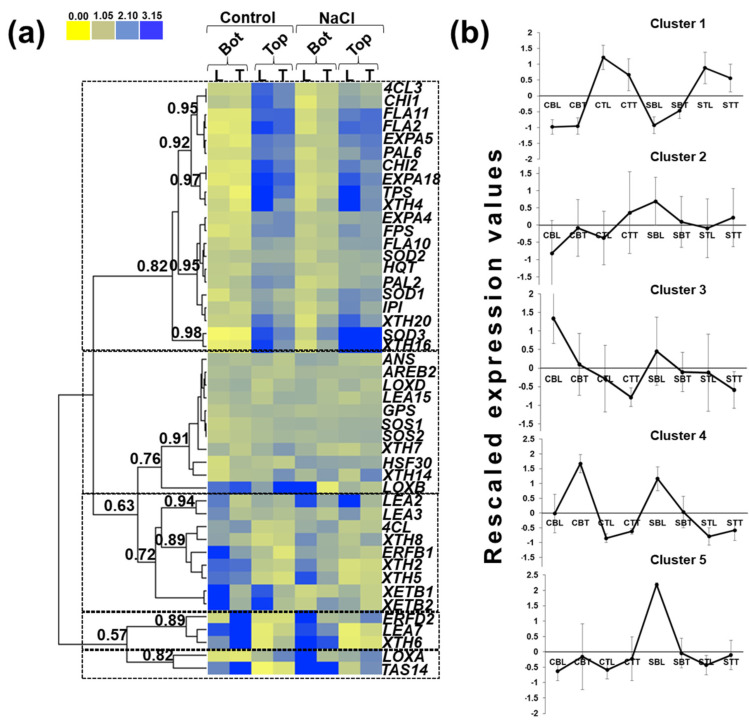
Gene expression analysis in Micro-Tom leaflets. (**a**) Heatmap hierarchical clustering (HC) of the gene expression data in control and stressed leaflets Micro-Tom exposed to 200 mM NaCl. The numbers refer to the Pearson’s correlation coefficients. The color bar indicates the pixel intensities which are directly proportional to the expression values. The dotted squares indicate the five clusters. CBL/SBL = control/salt-stressed bottom lateral leaflet; CBT/SBT = control/salt-stressed bottom terminal leaflet; CTL/STL = control/salt-stressed top lateral leaflet; CTT/STT = control/salt-stressed top terminal leaflet. (**b**) Rescaled expression values of each cluster. The rescaled values were calculated by subtracting from each expression value the average among the tissues and conditions and dividing by the standard deviation. *4CL* = 4-coumarate-CoA ligase, *ANS* = anthocyanidin synthase; *AREB* = ABA responsive element-binding protein; *CHI* = chalcone isomerase; *ERF* = ethylene-response factor; *EXPA* = expansin; *FPS* = farnesyl pyrophosphate synthase; *FLA* = fasciclin-like arabinogalactan protein; *GPS* = geranyl pyrophosphate synthase; *HQT* = hydroxycinnamoyl-CoA quinate transferase; *HSF* = heat shock factor; *IPI* = isopentenyl pyrophosphate isomerase; *LEA* = late embryogenesis abundant protein; *LOX* = lipoxygenase; *PA L*= phenylalanine ammonia lyase; *SOD* = superoxide dismutase; *SOS* = salt overly sensitive; *TAS14* = abscisic acid and environmental stress-inducible protein TAS14; *TPS* = terpene synthase; *XTH* = xyloglucan endo-transglycosylase/hydrolase; *XET* = xyloglucan endo-transglycosylase.

For ease of reading, the lateral and terminal leaflets sampled at the bottom and top under control condition or salt stress are abbreviated in Figure 7b, Figure S4 and Table S2 as follows: CBL control bottom lateral, CBT control bottom terminal, CTL control top lateral, CTT control top terminal, SBL salt-stressed bottom lateral, SBT salt-stressed bottom terminal, STL salt-stressed top lateral, STT salt-stressed top terminal (the positions and abbreviations are indicated in Figure S1).

The gene expression data are displayed in Figure 7a as a heatmap hierarchical clustering and in Figure S4 as bar chart. Figure 7b shows the rescaled expression values of each cluster, which were calculated by subtracting from each expression value the average among the tissues and conditions and dividing by the standard deviation.

By setting a Pearson’s coefficient threshold > 0.63, it was possible to distinguish five major clusters. Cluster 1 groups those genes whose expression was higher in younger leaflets sampled at the top compared to older ones from the bottom under control and salt stress conditions (Figure 7b).

The genes within cluster 2 showed overall lower expression values (as evidenced by the lower pixel intensities in Figure 7a) with an induction in bottom lateral leaflets under salt stress.

Cluster 3 comprises transcripts that were expressed at higher levels in lateral leaflets at the bottom and top regardless of salt stress.

Cluster 4 is composed of genes preferentially expressed in bottom terminal leaflets under control conditions and induced in bottom lateral leaflets under salt stress.

Finally, cluster 5 includes the two genes *LOXA* and *TAS14* which were induced in bottom lateral leaflets under salt stress.

#### 3.2.1. Genes Involved in the Phenylpropanoid Pathway

Several genes implicated in the phenylpropanoid pathway were found in cluster 1, such as *4CL3*, *PAL2*, *PAL6*, *HQT*, *CHI1* and *CHI2* (all gene acronyms are explained in the legend of Figure 7). *4CL3*, *PAL6*, *CHI1*, *CHI2* and *HQT* were upregulated at statistically significant levels in the younger leaflets sampled at the top compared to the older ones sampled at the bottom (Table S2). *CHI1* showed a statistically significant decrease in expression levels in the bottom lateral leaflets under salt stress condition (Table S2).

*PAL* is a well-known stress-responsive gene and its induction is responsible for lignification, as well as the synthesis of compounds such as flavonoids [35,41,42]. In salt-stressed plants, PAL activity was reported to increase under high salinity conditions (75 mM NaCl) compared to lower levels (i.e., 50 mM) and control [43]. However, no changes were observed in the present study in response to salinity. Several *PAL* isoforms exist in higher plants: for example, thale cress has 4 genes and tomato has at least 18 [44,45]. The genes here investigated may thus not directly participate in salt stress response in tomato leaflets but be instead involved in the biosynthesis of phenolic compounds under physiological conditions.

For *4CL*, which also partakes in the phenylpropanoid pathway and groups in cluster 3, the expression pattern showed non-significant differences between older and younger leaflets but indicated overall tendencies (higher values in bottom lateral and terminal leaflets of control plants). Downstream of PAL, 4CL plays a role both in lignin deposition and flavonoid synthesis. The *4CL* gene chosen in this study is the one likely associated with lignin biosynthesis [46]. Indeed, it was phylogenetically associated with class I clade, while those implicated in the synthesis of flavonoids were from class II [47].

#### 3.2.2. Genes Involved in Cell Wall Biosynthesis and Remodelling

Cell wall-related genes involved in expansion were also detected in the first cluster: three expansins *EXPA4*, *EXPA5*, *EXPA18*, three transcripts coding for fasciclin-like arabinogalactan proteins *FLA*2, *FLA10*, *FLA11*, as well as three xyloglucan endo-transglycosylase/hydrolase *XTH4*, *XTH20*, *XTH16*. The genes were expressed at statistically significant higher levels in top leaflets compared to bottom ones under control condition (Table S2); this is expected considering the need to expand of young tissues. Upon salt stress, the expression of expansins was either unaffected or tended to decrease. The tendency to decrease is indicative of the reported growth arrest in aerial organs of plants subjected to salt stress [35]. Expansins are genes involved in cell-wall remodelling by their loosening action on cellulose, but their exact mechanism remains poorly understood [48]. Their action has also been linked to stress adaptation, including salinity, since overexpression of an expansin in *Arabidopsis thaliana* conferred improved salt tolerance [49], while gene knock-out increased salt sensitivity [50].

*FLA* genes did not behave in the same way in bottom and top leaflets with respect to salinity, since *FLA11* and *FLA2* tended to be upregulated in bottom lateral and terminal leaflets of salt-stressed plants but downregulated in top lateral and terminal leaflets of NaCl-treated tomato. FLAs are involved in the maintenance of the continuum between plasma membrane and cell wall. Their expression was shown to be affected by salt stress in other plant species, such as textile hemp [35].

*XET*/*XTH* are genes coding for xyloglucan endo-transglucosylase/hydrolase, enzymes that play an important role in cell wall remodelling by acting on xyloglucans, the chief hemicellulose in primary cell walls. Xyloglucans bridge cellulose microfibrils and thus contribute to the mechanical properties of the cell walls and to morphogenesis [51,52]. More specifically, xyloglucan chains may mediate the contact between cellulose microfibrils and determine “biomechanical hotspots”, i.e., limited regions of wall extensibility [52].

XTHs have both xyloglucan endo-transglucosylase (XET, i.e., they cut and rejoin xyloglucan chains) and xyloglucan endo-hydrolase (XEH, i.e., they hydrolyze xyloglucan) activities [53,54,55,56]. *XTH4*, *XTH16*, *XTH20* were all expressed at statistically significant higher levels in top lateral leaflets under control condition (Figure S4 and Table S2). *XTH5*, *XTH8* and *XTH6* were, however, preferentially expressed in bottom lateral leaflets compared to terminal ones under control condition (they belong to cluster 3 and 4, Figure 7a) and tended to be induced in bottom lateral leaflets under salt stress (Figure S4). A link between *XTH* expression/activity and salt tolerance was previously demonstrated: the overexpression of *CaXTH3* (from *Capsicum annuum*) in tomato resulted in an increased tolerance to salt stress, as evidenced by the higher levels of chlorophyll, as well as the reduced transpiration by means of increased stomatal cell wall remodelling [57].

#### 3.2.3. Genes Involved in Volatile Emission and Stress Response

Genes implicated in volatile organic compounds’ (VOCs) synthesis (*TPS*, *FPS*, *IPI*) were all expressed at statistically significant higher levels in top lateral leaflets under control condition (Table S2). Terpene synthase (*TPS*) and farnesyl pyrophosphate synthase (*FPS*) followed a trend towards downregulation by salinity (Figure S4). These results are in agreement with those reported previously by Zhang and colleagues [43], who measured a downregulation of volatile emission-related genes under salt stress, while the produced amount of some VOCs, as well as their emission profile, changed with osmotic stress.

Superoxide dismutase genes (*SOD*s) code for proteins involved in the enzymatic response to ROS. Three *SOD* genes were here monitored and they all grouped in the first cluster (Figure 7a). They were expressed at statistically significant higher levels in top lateral leaflets under control and salt stress conditions (Table S2). Moreover, *SOD3* tended to be induced in lateral and terminal leaflets at the bottom and top under salt stress. Due to their ROS-detoxifying function, *SOD*s are generally induced by stress [58], but their increased activity is also a sign of stress alleviation [59]. However, it was previously reported that *SOD*s are a group of early-responsive genes whose expression shows a peak shortly after stress application due to the occurrence of salt-shock [60]. Therefore, it is legitimate to speculate that *SOD*s expressions might have been higher if sampling had been performed earlier. Long-term stress application may affect the expression of ion toxicity-related genes rather than osmotic stress-related transcripts [61].

Genes related to stress signaling/response, more specifically responding to ethylene, a plant hormone mediating several aspects of the (a)biotic stress response (*ERFB1*) and belonging to the LEA family (*LEA7*) showed a significant upregulation in bottom terminal leaflets compared to top terminal ones under control condition (Table S2). The dehydrin *TAS14* tended to be induced in salt-treated bottom lateral leaflets (Figure S4); in tomato, *TAS14* was shown to be induced by salinity and osmotic stress, as well as abscisic acid and its overexpression led to plants capable of withstanding drought and salinity in the long term, without any impact on plant growth under control conditions [62].

The genes coding for late embryogenesis abundant (LEA) proteins accumulated in response to water deficit in many organisms such as tomato, where 27 *LEA* genes were found and classified in several groups, namely dehydrins, *LEA1*, *LEA2*, *LEA3*, *LEA4*, *LEA5* and seed maturation proteins-SMPs [63]. These proteins were shown to have membrane stabilizing and maintenance functions [64], as well as ion sequestration properties [65]. They were moreover shown to be implicated in general (a)biotic stress response [66].

*LEA15* showed a significant increase in expression in bottom lateral leaflets under salt stress (Table S2). This is in agreement with the reported increased expression of *LEA15* in response to salinity and drought in tomato seedlings [63]. It should be noted that this gene was expressed in both vegetative and reproductive organs of tomato and could thus represent an important gene involved in both physiological and stress-related conditions.

Salt overly sensitive (*SOS*) genes are involved in salinity tolerance through the regulation of Na^+^ extrusion from the roots, the active loading of Na^+^ into the xylem and the compartmentalization of Na^+^/K^+^ [67]. In the present study, however, no significant differences were observed for *SOS1* and *SOS2*. The reasons for this can be: 1) the expression of *SOS* in Micro-Tom may show significant changes at earlier time-points after salt application, or 2) tissues other than leaflets (i.e., flower, fruits, roots) may show statistically significant differences in expression.

Lipoxygenases (LOXs) are enzymes catalyzing the key step of lipid peroxidation to produce compounds such as oxylipins, but they are also indicative of membrane damage [68]. *LOX*s showed different expression patterns. *LOXA*, which was expressed at higher levels in top terminal leaflets rather than bottom terminal ones under control condition, was significantly higher in salt-treated bottom lateral leaflets compared to control ones (Table S2). *LOXB* and *LOXD* grouped in another cluster (Figure 7a) and did not show any statistically significant differences in expression under the experimental conditions studied. *LOXA* and *LOXD* are involved in a different metabolic pathway, since they are implicated in 9-LOX and 13-LOX synthesis, respectively, which are two molecules related with the response to herbivores [69]. LOX activity was reported to be higher in salt-stressed tomato plants (exposed to 75 mM NaCl) compared to control plants [43]. Similarly, *LOX*s expression was induced by salinity and rose with silicon nanoparticles application under salt stress [70].

The heat shock factor (*HSF*) gene family contains 26 highly conserved members in *S. lycopersicum* and plays an important role in high-temperature stress response [71]. The gene coding for the heat shock transcription factor *HSF30* was significantly higher in expression in salt-treated bottom lateral leaflets compared to control ones (Figure 7 cluster 2 and Table S2). In *S. chilense*, *HSF30* showed a log2 fold change > 5 upon salt stress, thereby confirming its role in salinity tolerance in this wild species [9]

## 4. Conclusions

The results obtained in this case study focused on Micro-Tom showed that salt stress impacted the growth of plants and differences were observed both at the microscopic and gene expression levels. Older leaflets sampled from the bottom of the stem showed stronger stress signs as compared to younger ones at the top, namely a less developed xylem tissue and modifications of the palisade parenchyma’s structure. From the rescaled expression values, it was apparent that, in most clusters, gene expression was higher in salt-stressed bottom leaflets compared to their respective control. Furthermore, stress-related genes such as *LEA15, HSF30*, *LOXA* and *TAS14* were, by trend, induced in response to the applied stress in bottom leaflets. For future molecular studies on Micro-Tom, it is therefore proposed to focus on older leaflets located at the bottom of the stem when investigating salt stress response in this tomato model.

## Data Availability

The data presented are all made available in this study.

## Supplementary Materials

The following are available online at https://www.mdpi.com/article/10.3390/genes12081165/s1, Figure S1: Schematic representation of the sampling procedure, Figure S2: Measures of the xylem vessels’ (a,b) area and thickness (c,d) in bottom and top leaflets, Figure S3: Measures of the intercellular spaces in the spongy parenchyma of bottom (a–d) bottom and top leaflets (e–h), Figure S4: Graph of the expression data shown in Figure 7a, Table S1: List of primers used in the study with details of amplicons’ size and amplification efficiency, Table S2: Statistical significance of the expression data shown in Figure S2.
