# Supplementary material for: The Effects of Salinity on the Anatomy and Gene Expression Patterns in Leaflets of Tomato cv. Micro-Tom
Source: Genes (Basel). 2021 Jul 29;12(8):1165. doi: 10.3390/genes12081165 (PMC8392013; doi:10.3390/genes12081165)
Supplement: Supplementary file 1 [file genes-12-01165-s001.zip › Supplementary Materials.pdf]

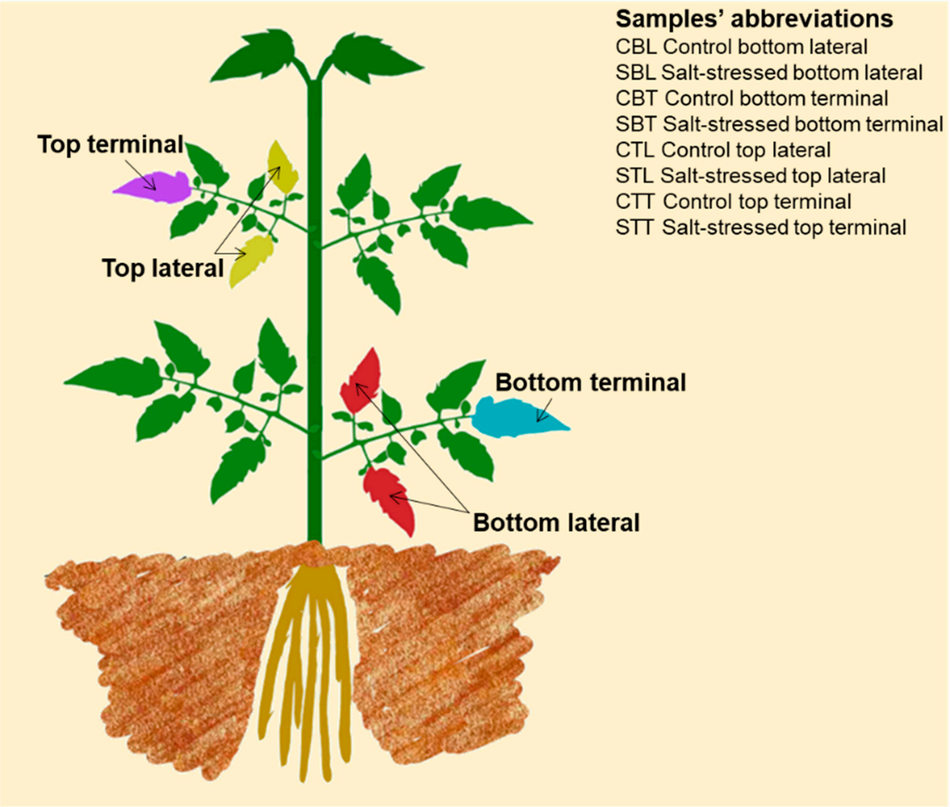

Figure S1. Schematic representation of the sampling procedure and samples' abbreviations used. The sampled leaflets are colored and indicated with arrows. Purple: top terminal leaflet, yellow: top lateral leaflets, aquamarine: bottom terminal leaflet, red: bottom lateral leaflets.

Table S1. List of primers used in the study with details of amplicons' size and amplification efficiency.

Gene	Solyc. code	Primer Fwd (5'→3')	Primer Rev (5'→3')	Amplic. size (bp)	Amplif. efficiency
4CL	Solyc12g094520.1	CGTAGACCAATTCCGTTTCC	GATTTTCCCGCTATGTGCTC	81	1.90
4CL3	Solyc03g097030.2	TCATGTGGCTCTGTGATTCG	CAGTGGCTTCATCGTCATTG	145	1.92
ANS	Solyc08g080040	AAGTTGGAGGCATGGAAGAC	GTTCTGGTTGGGGACATTTG	72	1.92
AP2C	Solyc02g093150.2	AAACGGAGACACTTGGCAAC	ACGGCGGCTCTTCTTAATAG	86	1.90
AREB2	Solyc11g044560.1	TGCAGAGACAGGGTTCTTTG	CTGCCCAAAGTTTGATCCTC	134	1.92
CHI1	Solyc05g052240	GAAGAAGAGGAAGAAGCACTCG	ATCTTTGCACTCCCAGAAGC	70	1.93
CHI2	Solyc05g010320	GGAGTTGGAACATTCAGTCG	CGTCAAGGGCAAGATCATAG	91	1.90
Cyclo	Solyc07g025390.2	GCTAAGAACGCTGGACCTAATG	TGGGTGTGCCTTTCTGAATG	183	1.90
ERFB1	Solyc05g052040.1	GAATGATGACGGAATTGTAATGAAGA	TTCCACAATCCCAAATTGAAGA	101	1.93
ERFD2	Solyc12g056590.1	CATAAAGCCGCCAGAGTTTG	TGACAATCTGGCGTTCTCAG	132	2.00
EXPA18	Solyc06g076220.2	GTTGTGGAGCGTGTTTTGAG	GTTTGGGAGAGCAAAGTTGG	119	1.91
EXPA4	Solyc09g010860.2	GATGGCAAATGGTGTCTTCC	CCGTCATCGTTTGGTAAAGC	92	1.92
EXPA5	Solyc02g088100.2	CGCCCTCATTTTGATCTCTC	AATTCCTCCGCTTCTTCTGC	111	1.93
FLA10	Solyc10g005960.1	AACGGTGTTTTGTCCTGGAG	CCCATGATACTCAAGCAACG	100	1.92
FLA11	Solyc11g069250.1	CCTTCTGGTCCAACAAACATC	CATCCCATTCCCTTGATTTG	138	2.03
FLA2	Solyc07g048090.1	AGTCCCTTTGCCTTTTCCAC	ATAAGTTCCCTCCAGCCAAG	105	1.99
FPS	Solyc12g015860.1	CATACACGCCGAGGTCAAA	GCAGCAATCATGCCAACCTT	59	1.91

FUL1	Solyc06g069430.2	GTTTTGCCACAACAACTGGACTC	CTTGCTGCTGTGAAGAACTACC	106	1.91
GPS	Solyc08g023470.2	TGTGATGGGAAATAAGCTAGCTGTA	ACACATGCTCGGGAAAGCA	60	1.93
HQT	Solyc07g005760.2	CCAATGGCTGGAAGATTAGG	GCCTCAACAAACAAAACACC	83	1.90
HSF30	Solyc08g062960.2	CCTGACAGATGGGAATTTGC	CAAGCACCAGATCCTTGTTG	119	1.90
IPI	Solyc04g056390.2	TGTCCCAGTTGACCAGTTCATT	TTCCCGTCAGATGGAGCTTT	63	1.98
LEA2	Solyc01g095140.2	GGTCTCGCTATTGATCTTCCAG	CTTCTTGTTTGTCACCGTTCC	112	1.98
LEA3	Solyc01g095150.2	AGGGACATTGGAGGAGATTG	AGAGGGGAATGGTGATGTTG	94	1.96
LEA7	Solyc02g084840.2	AAATTGCCAGGAGGTCACAG	AGTGCCTTCCATGCCATAAC	81	2.00
LEA15	Solyc08g015690.2	GGGCTTCATCCAGGATATTG	GGGTTTGGGTTCTTCACAAG	146	1.94
LOXA	Solyc08g014000.2	GGCTGAAATGGAAACACCAC	GGATACTGCCCGAAATTGAC	99	1.97
LOXB	Solyc01g099190.2	GTCTTTGGGTGGAATTGTGG	GCCATCAACAACTGAAGCAC	127	1.92
LOXD	Solyc03g122340.2	GCTTCAGGTTTTTCCACCAG	GCATCCAAAGCCTCTTGAAC	126	1.93
PAL2	Solyc05g056170.2	AGTGATTGGGTGATGGAAGG	TTTTGTAGGGCTGCACCTTG	110	1.91
PAL6	Solyc10g086180.1	TGAGACGTTGAATGCTGAGG	AGCCAAACCAGAACCAACAG	121	1.89
SAND	Solyc03g115810.2	GATTCAGGGGTTCTGTTTGC	TCATCAGGGTGAAGAGATGC	89	2.01
SOD1	Solyc01g067740.2	AACCTGGACTTCATGGCTTC	CCAGCAGGATTGTAATGTGG	88	1.98
SOD2	Solyc03g062890.2	GCAATCCAATGGTGTTACCC	AGTGGAATTGCAGCCGTTAG	112	1.96
SOD3	Solyc11g066390.1	CTCCTGGAGATGAAATCCGT	AAGTGCTCGTCCAACAACTG	134	1.90
SOS1	Solyc01g005020.2	AAGGAAAGCGAGGAAGAAGG	TCGGAGAACCTGAGGAAATG	78	1.90
SOS2	Solyc12g009570.1	CAAGAATTGAGGGGGTAACG	AAAAGGGAAGGGGCTACTTG	82	1.90
TAS14	Solyc02g084850.2	CGGCAATCAAGACCAAATG	TCCATATTCTCCACCAGTGC	145	1.93
TPS	Solyc12g006570.1	CAACTCCCAACTTGCACAACA	CCCTCACAATGCCATCTTTAATT	68	1.93
XETB1	Solyc12g017240.1	GCAGCAGCTATCTGTTCTTGC	AGGTAACCGTTGCATCTTGG	83	1.93
XETB2	Solyc07g056000.2	GTTCAACAACCACGAAGCAC	TGTGTAGCCCAATCATCAGC	96	1.89
XTH14	Solyc09g008320.2	AATCTCTGGGGTCCTAATCACC	CCACTGCCTGAATTTCTATCAAG	77	1.91
XTH16	Solyc07g052980.2	GCTTGTTGAAGGTGACTCTGC	ATTGTGGGTTGGTCCATCTG	70	1.92
XTH2	Solyc07g009380.2	CGAAAACGGGCAACTTCTTA	CTGACCCCACCGAAGATAAA	164	1.92
XTH20	Solyc07g006870.2	GCACTGTCACCGCATACTATTTG	GCTCTCTATCACCTTTGCCTTG	138	2.08
XTH4	Solyc11g065600.1	TGATGTGCCTTTTTGGAACA	AGCAGTGACAACACCAGCAG	199	1.91
XTH5	Solyc01g081060.2	GTGCGAGATTGATCCAGTTG	GATGACGATGTCCGTGGTG	86	1.96
XTH6	Solyc11g066270.1	CCCCTTCACCTGGCTACTATC	TGATGTTGAGGACCCCAAAG	88	1.91
XTH7	Solyc02g091920.2	ATGTCCACGGAAAAGGTGAC	GAGAACACGGCTTGATGATG	109	1.91
XTH8	Solyc04g008210.1	CCTTCATGGCTTATTCAGTGC	AAATGCAACCACCACTCCAG	70	1.92

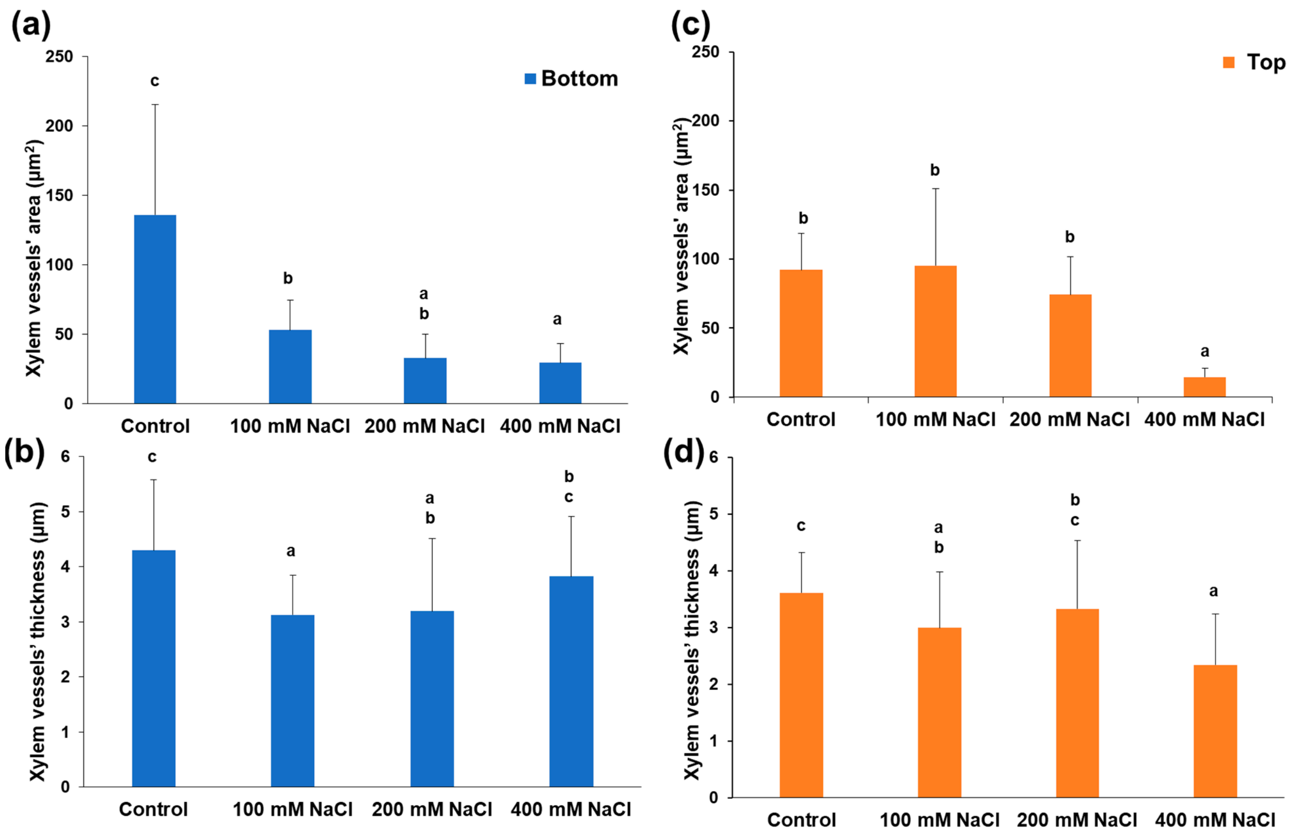

Figure S2. Measures of the xylem vessels' (a-b) area and thickness (c-d) in bottom and top leaflets. Bottom area [$F(3,52)=21.94$, p-value=0.000]; Top area [$X^2(3)=23.24$, p-value=0.000]; Bottom thickness [$F(3,124)=8.46$, p-value=0.000]; Top thickness [$X^2(3)=17.46$, p-value = 0.000]. A compact letter display [26] is used to indicate the significance between groups of samples.

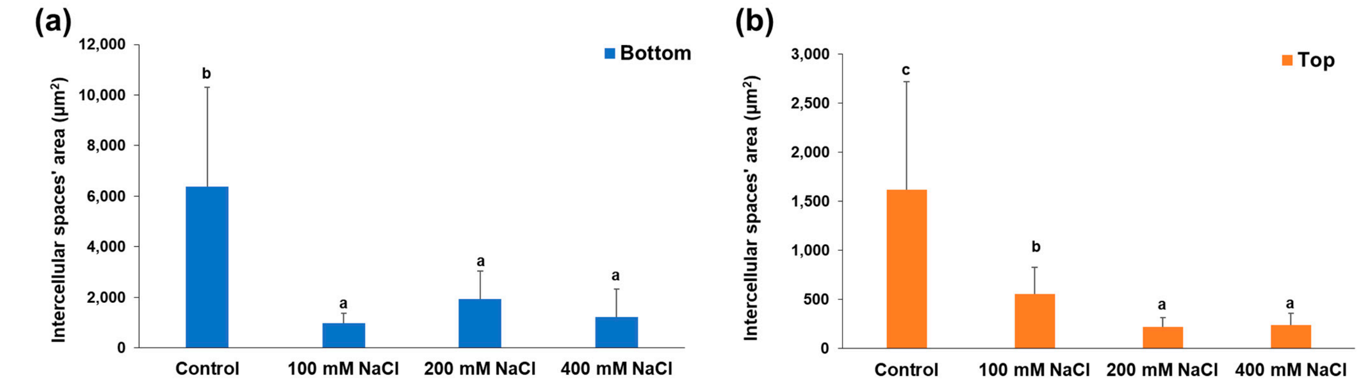

Figure S3. Measures of the intercellular spaces in the spongy parenchyma of bottom (a-d) bottom and top leaflets (e-h). Bottom intercellular spaces [$X^2(3)=15.42$, p-value=0.001]; Top intercellular spaces [$F(3,35)=22.36$, p-value=0.000]. A compact letter display [26] is used to indicate the significance between groups of samples.

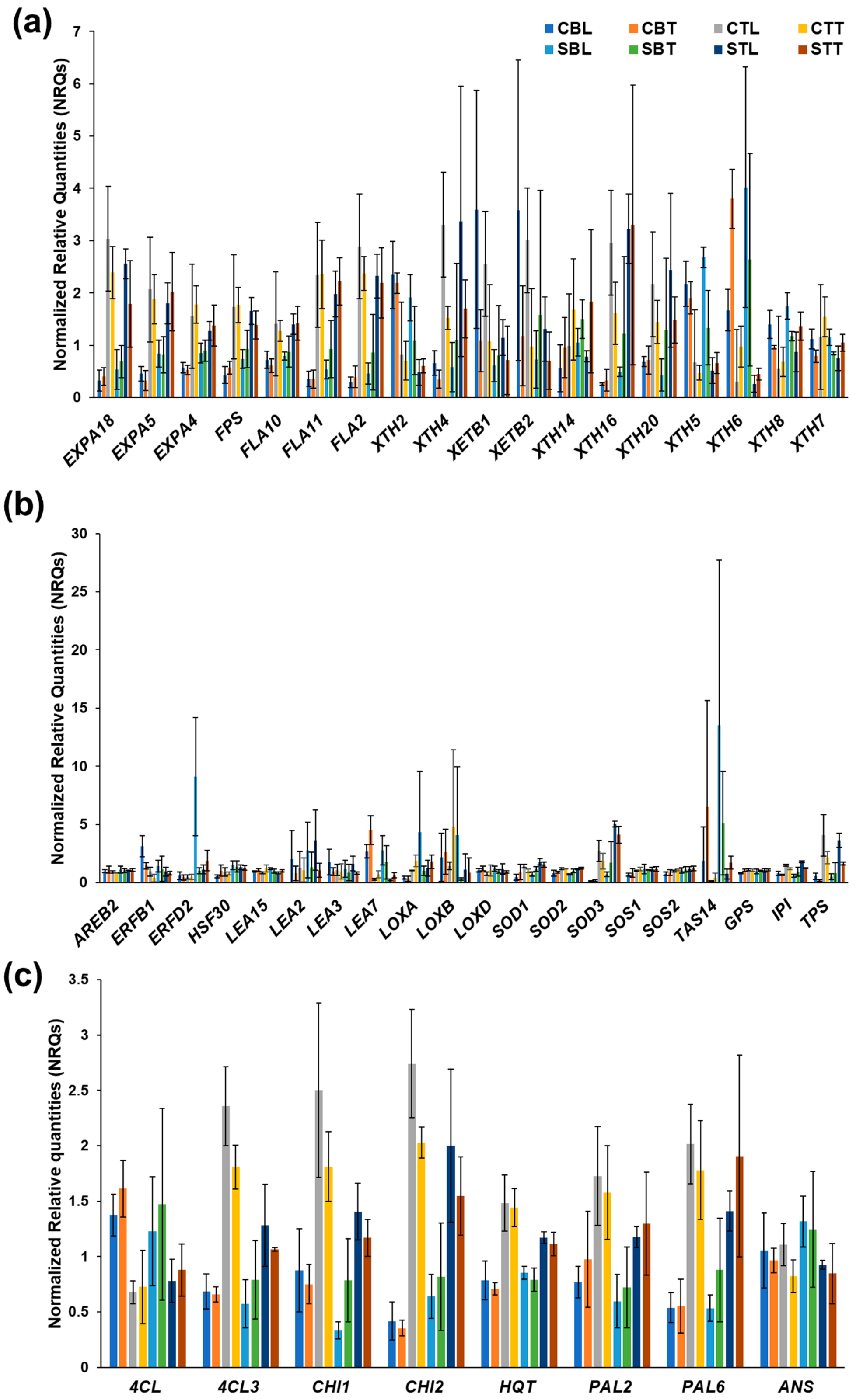

Figure S4. Graph of the expression data shown in Figure 7a. The values are normalized relative quantities (NRQs) ± standard deviation (n=3). The statistical significance is provided in Table S2. CBL/SBL= control/salt-stressed bottom lateral leaflet; CBT/SBT= control/salt-stressed bottom terminal leaflet; CTL/STL= control/salt-stressed top lateral leaflet; CTT/STT= control/salt-stressed top terminal leaflet.

Table S2. Statistical significance of the expression data shown in Figure S2. The character in italics refer to the letters assigned with the non-parametric test (Kruskal-Wallis followed by Dunn's post-hoc test), while the others indicate those assigned with the parametric test (one-way ANOVA followed by Tukey's post-hoc test). A compact letter display [26] is used to indicate the significance between groups of samples.

	**CBL**	**CBT**	**CTL**	**CTT**	**SBL**	**SBT**	**STL**	**STT**
4CL3	*a*	*a*	*b*	*b*	*a*	*a*	*ab*	*ab*
CHI1	bc	ab	d	cd	a	ab	bcd	bcd
FLA11	a	a	b	b	a	ab	b	b
FLA2	*a*	*ab*	*d*	*cd*	*abc*	*abc*	*cd*	*bcd*
EXPA5	a	a	b	b	ab	ab	b	b
PAL6	a	a	b	b	a	ab	b	b
CHI2	*a*	*a*	*c*	*bc*	*ab*	*ab*	*bc*	*abc*
EXPA18	*a*	*a*	*b*	*b*	*a*	*a*	*b*	*ab*
TPS	*ab*	*a*	*c*	*bc*	*ab*	*ab*	*c*	*abc*
XTH4	*a*	*a*	*b*	*ab*	*a*	*ab*	*b*	*ab*
EXPA4	a	a	c	c	ab	ab	bc	bc
FPS	a	ab	d	d	abc	bcd	d	cd
FLA10	a	a	c	bc	ab	abc	c	c
SOD2	*ab*	*abc*	*cd*	*d*	*a*	*abcd*	*bcd*	*c*
HQT	ab	a	d	d	abc	ab	cd	bcd
PAL2	abc	abc	c	bc	a	ab	abc	abc
SOD1	*a*	*abc*	*bcd*	*abc*	*ab*	*abcd*	*d*	*cd*
IPI	*ab*	*ab*	*cd*	*abcd*	*a*	*abc*	*d*	*bcd*
XTH20	*ab*	*ab*	*c*	*abc*	*a*	*abc*	*bc*	*abc*
SOD3	*a*	*ab*	*bcd*	*abcd*	*abc*	*abcd*	*d*	*cd*
XTH16	*a*	*a*	*b*	*ab*	*ab*	*ab*	*b*	*b*
ANS	*a*	*a*	*a*	*a*	*a*	*a*	*a*	*a*
AREB2	a	a	a	a	a	a	a	a
LOXD	a	a	a	a	a	a	a	a
LEA15	*ab*	*bc*	*a*	*bc*	*c*	*abc*	*a*	*abc*
GPS	*a*	*a*	*a*	*a*	*a*	*a*	*a*	*a*
SOS1	a	a	a	a	a	a	a	a
SOS2	a	a	a	a	a	a	a	a
XTH7	a	a	a	a	a	a	a	a
HSF30	a	ab	ab	ab	b	b	b	ab
XTH14	a	a	a	a	a	a	a	a
LOXB	*a*	*a*	*a*	*a*	*a*	*a*	*a*	*a*
LEA2	a	a	a	a	a	a	a	a
LEA3	a	a	a	a	a	a	a	a
4CL	a	a	a	a	a	a	a	a
XTH8	bc	abc	a	ab	c	bc	abc	bc
ERFB1	b	b	ab	a	ab	ab	ab	ab
XTH2	c	bc	abc	abc	bc	abc	a	ab
XTH5	*bc*	*abc*	*a*	*a*	*c*	*abc*	*a*	*ab*
XETB1	*a*	*a*	*a*	*a*	*a*	*a*	*a*	*a*
XETB2	a	a	a	a	a	a	a	a
ERFD2	*a*	*a*	*a*	*a*	*a*	*a*	*a*	*a*
LEA7	c	c	a	ab	c	bc	a	ab

XTH6	***abc***	***c***	***ab***	***abc***	***bc***	***bc***	***a***	***ab***
LOXA	***a***	***a***	***ab***	***bc***	***bc***	***abc***	***abc***	***c***
TAS14	ab	ab	a	ab	b	ab	ab	ab

4CL [F(7,16)=3.13, *p*-value=0.028], *AREB2* [F(7,16)=0.79, *p*-value=0.605], *CHI1* [F(7,16)=12.58, *p*-value=0.000], *ERFB1* [F(7,16)=4.63, *p*-value=0.005], *EXPA5* [F(7,16)=12.74, *p*-value=0.000], *EXPA4* [F(7,16)=15.47, *p-value*=0.000], *FDS* [F(7,16)=14.64, *p-value*=0.000], *FLA10* [F(7,16)=9.94, *p*-value=0.000], *FLA11* [F(7,16)=13.53, *p*-value=0.000], *HQT* [F(7,16)=14.45, *p*-value=0.000], *HSF30* [F(7,16)=3.79, *p*-value=0.013], *LEA2* [F(7,16)=0.53, *p*-value=0.802], *LEA3* [F(7,16)=0.88 *p*-value=0.542], *LEA7* [F(7,16)=15.59, *p*-value=0.000], *LOXD* [F(7,16)=0.96, *p*-value=0.493], *PAL2* [F(7,16)=4.42, *p*-value=0.007], *PAL6* [F(7,16)=9.75, *p*-value=0.000], *SOS1* [F(7,16)=2.50, *p*-value=0.061], *SOS2* [F(7,16)=2.09, *p*-value=0.105], *TAS14* [F(7,16)=3.52, *p*-value=0.018], *XTH2* [F(7,16)=5.88, *p*-value=0.002], *XETB2* [F(7,16)=1.33, *p*-value=0.299], *XTH14* [F(7,16)=1.99, *p*-value=0.119], *XTH8* [F(7,16)=6.38, *p*-value=0.001], *XTH7* [F(7,16)=2.31, *p*-value=0.078], *4CL3* [X^2(7)=18.49, *p*-value=0.010], *ANS* [X^2(7)=8.71, *p*-value=0.274], *CHI2* [X^2(7)=20.03, *p*-value=0.006], *ERFD2* [X^2(7)=10.88, *p*-value=0.144], *EXPA18* [X^2(7)=20.17, *p*-value=0.005], *FLA2* [X^2(7)=19.16, *p*-value=0.008], *GPS* [X^2(7)=8.57, *p*-value=0.285], *IPI* [X^2(7)=19.53, *p*-value=0.007], *LEA15* [X^2(7)=15.37, *p*-value=0.032], *LOXA* [X^2(7)=17.13, *p*-value=0.017], *LOXB* [X^2(7)=6.97, *p*-value=0.432], *SOD1* [X^2(7)=17.08, *p*-value=0.017], *SOD2* [X^2(7)=19.09, *p*-value=0.008], *SOD3* [X^2(7)=20.47, *p*-value=0.005], *TPS* [X^2(7)=20.03, *p*-value=0.006], *XTH4* [X^2(7)=15.00, *p*-value=0.036], *XETB1* [X^2(7)=12.41, *p*-value=0.088], *XTH16* [X^2(7)=17.19, *p*-value=0.016], *XTH20* [X^2(7)=14.23, *p*-value=0.047], *XTH5* [X^2(7)=17.99, *p*-value=0.012], *XTH6* [X^2(7)=17.41, *p*-value=0.015].
