# Supplementary figures and images for: The Effects of Salinity on the Anatomy and Gene Expression Patterns in Leaflets of Tomato cv. Micro-Tom
Source: Genes (Basel). 2021 Jul 29;12(8):1165. doi: 10.3390/genes12081165 (PMC8392013; doi:10.3390/genes12081165)

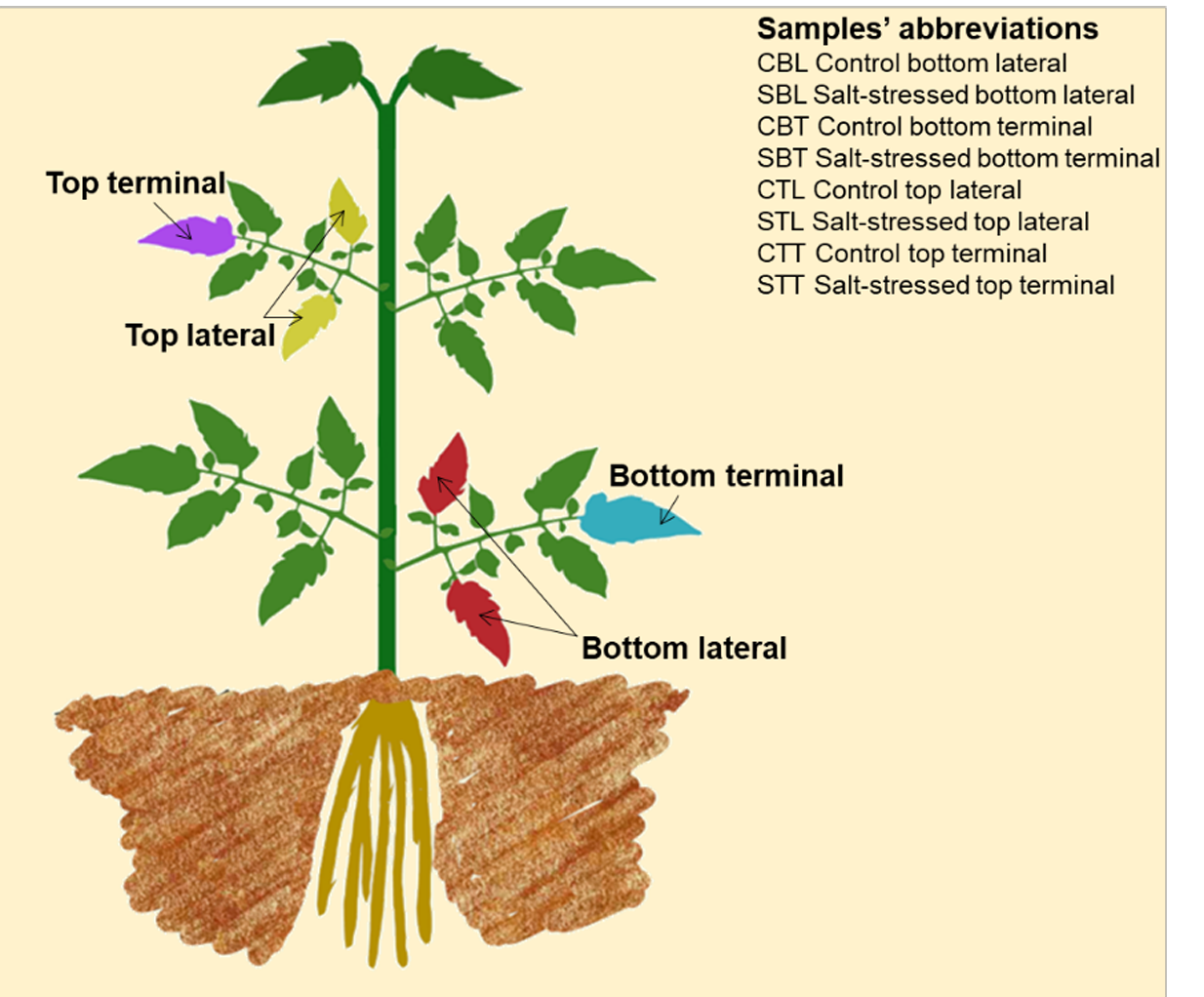

Supplement: Supplementary file 1 [file genes-12-01165-s001.zip › Figure S1.tif]
